# Quantitative Analysis of Lumbar Disc Bulging in Patients with Lumbar Spinal Stenosis: Implication for Surgical Outcomes of Decompression Surgery

**DOI:** 10.3390/jcm12196172

**Published:** 2023-09-24

**Authors:** Koji Akeda, Takahiro Hasegawa, Yusuke Togo, Kento Watanabe, Koki Kawaguchi, Junichi Yamada, Norihiko Takegami, Tatsuhiko Fujiwara, Akihiro Sudo

**Affiliations:** Department of Orthopaedic Surgery, Mie University Graduate School of Medicine, Tsu 514-8507, Japan; hasegawa-t@med.mie-u.ac.jp (T.H.); fd2-7212@med.mie-u.ac.jp (Y.T.); 05190326yui@med.mie-u.ac.jp (K.W.); k-kawaguchi@med.mie-u.ac.jp (K.K.); yamada-j@med.mie-u.ac.jp (J.Y.); n-takegami@med.mie-u.ac.jp (N.T.); tatsuhiko-f@med.mie-u.ac.jp (T.F.); a-sudou@med.mie-u.ac.jp (A.S.)

**Keywords:** disc bulging, lumbar spinal stenosis, CT, MRI, patient-reported outcome measures

## Abstract

This study aimed to quantitatively assess disc bulging using computed tomography (CT) in patients with lumbar spinal stenosis (LSS) and to examine whether disc bulging affects the surgical outcomes of patients with LSS after posterior decompression surgery. Sixty-three patients who underwent posterior decompression surgery for LSS were included. The extent of disc bulging was evaluated as the percentage of the extended area of the disc against the endplate area (%EAD) on axial CT images. The participants completed the following clinical outcome assessments (COAs) preoperatively and 12 months postoperatively: the JOA Back Pain Evaluation Questionnaire (JOABPEQ), Oswestry Disability Index (ODI), and Roland–Morris Disability Questionnaire (RDQ). The mean %EAD of 315 intervertebral discs was 18.9 ± 8.0. The %EAD was highest at L4/L5, followed by L3/L4, L2/L3, L1/L2, and L5/S1. The %EAD of the surgical level showed no significant correlation with all the preoperative COAs, but it had significant correlation with lumbar function, walking ability, social function domains of the JOABPEQ, ODI score, and RDQ score 12 months postoperatively. %EAD was significantly associated with the postoperative score in the walking ability domain of the JOABPEQ. %EAD affects postoperative clinical outcomes, including low back pain-related quality of life after decompression surgery.

## 1. Introduction

Lumbar intervertebral disc (IVD) degeneration is associated with the development of low back pain (LBP) and lumbar degenerative diseases, including lumbar spinal stenosis (LSS), and is a clinically important problem [1]. The progression of IVD degeneration leads to structural changes in IVD tissues, resulting in disc bulging [2]. The presence of disc tissue extending beyond the edges of the ring apophyses, throughout the circumference of the disc, is called “disc bulging” and is not considered a form of herniation according to the nomenclature and classification of lumbar disc pathology [3]. Asymmetric bulging of disc tissue greater than 25% of the disc circumference is usually recognized as a form of “disc bulging” but not disc herniation [3]. However, its precise definition and clinical significance remain unclear.

LSS is characterized by the narrowing of the spinal canal caused by degenerative changes in the lumbar structures with compression of neural structures [4]. Disc bulging (disc degeneration), thickening of the ligamentum flavum, and facet hypertrophy contribute to the narrowing of the spinal canal, which is greatest at the level of the disc segments. When conservative treatments are less effective, surgical treatments, including posterior decomposition of the neural tissue with or without fusion, show good clinical outcomes [4,5]. However, whether disc bulging affects the neurological symptoms and surgical outcomes after posterior decompression surgery in patients with LSS remains unclear.

This preliminary study aimed to quantitatively assess the morphometry of disc bulging using computed tomography (CT) in patients with LSS and to examine the effects of disc bulging on the surgical outcomes of patients with LSS after posterior decompression surgery.

## 2. Materials and Methods

### 2.1. Participants

Sixty-three patients (51 men and 12 women; average age, 69.9 ± 8.6 years) who underwent posterior decompression surgery for lumbar canal stenosis from August 2018 to July 2022 were included in this study. Anthropometric measurements, including body height, body weight, and body mass index (BMI), were obtained from the patients.

### 2.2. Computed Tomography Evaluation

**Figure 1:**
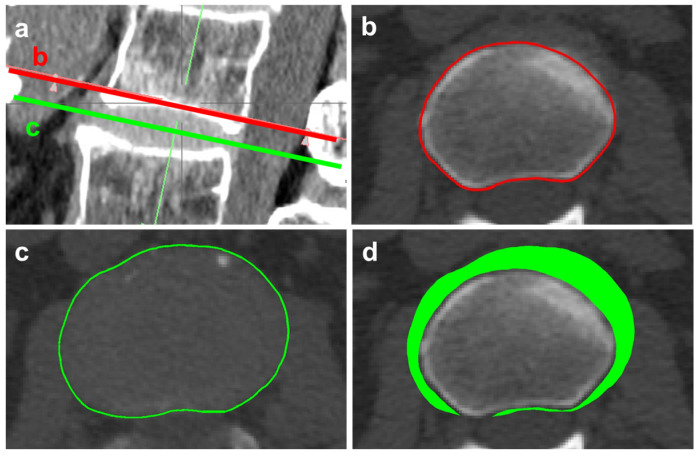
Measurement of % extended area of disc (EAD). Sagittal reconstruction image of lumbar CT (**a**). The cross-sectional area of endplates (endplate area) enclosed by red line was determined at the concave apex of the superior endplate of each disc level (**b**). The cross-sectional area of IVDs (disc area) enclosed by green line was determined at the midline between the superior and inferior endplates (**c**). Extended area of disc (EAD) filled in green was calculated as the subtraction of the endplate area from the disc area (**d**). %EAD was calculated as follows: (disc area-endplate area)/endplate area ×100.

The patients underwent CT preoperatively using a SOMATOM Definition Flash CT scanner (Siemens, Munich, Germany) with a 1.0 mm slice thickness and scan resolutions ranging from 260 to 310 μm. An axial CT-multiplanar reconstruction (MPR) was performed at all lumbar intervertebral levels (from L1/L2 to L5/S1) using a clinical image analysis software (AquariusNet Viewer version 4.4.13; P2, TeraRecon, Durham, NC, USA). The cross-sectional area of the IVDs (disc area) was determined at the midline between the superior and interior endplates. The regions of interest (ROIs) of the disc area were set along the outer margin of the disc area identified on the axial CT images, adjusting the window level to 40 (window width, 300). The cross-sectional area of the endplate (endplate area) was determined at the concave apex of the superior endplate at each disc level (Figure 1a). The ROIs of the endplate area were set along the outer margin of the endplate area identified on axial CT images in the bone window setting. The extended area of the disc (EAD) was calculated by subtracting the endplate area from the disc area (Figure 1). %EAD was calculated as follows: (disc area − endplate area)/endplate area × 100. The patients were divided into the following two groups: %EAD <30 and ≥30.

### 2.3. Magnetic Resonance Imaging Evaluation

**Figure 2:**
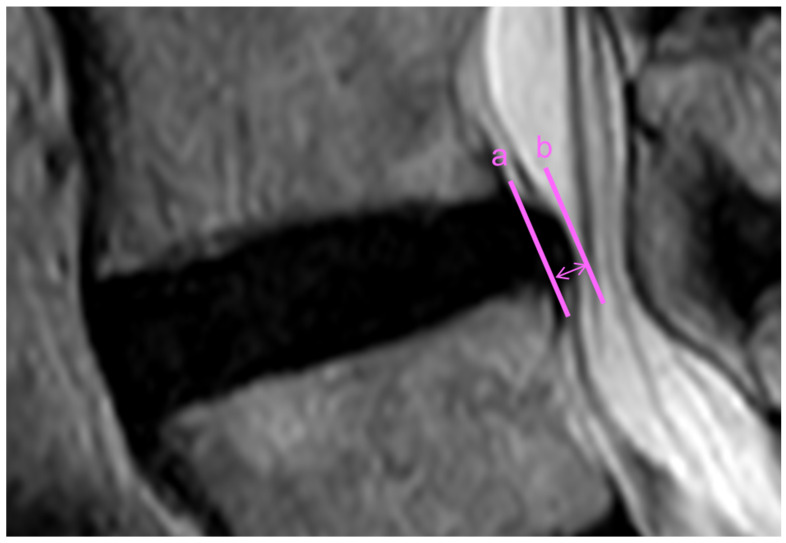
Measurement of disc bulging length (DBL). DBL (two-headed arrow), defined as the distance from the posterior end of the bulging disc (line a) to the posterior wall of the lumbar vertebra (line b), is measured on midsagittal magnetic resonance imaging (T2-weighted image).

The cross-sectional area of the IVDs (disc area) was determined at the midline between the superior and interior endplates. The ROIs of the disc area were set along the outer margin of the disc area identified on axial MRI (T2-weighted images). Disc bulging length (DBL), defined as the distance from the posterior end of the bulging disc to the posterior wall of the lumbar vertebra, was measured using midsagittal MRI (T2-weighted image) (Figure 2). Disc height was evaluated using the disc height index (DHI) on MRI, as previously reported [6]. The cross-sectional area of the thecal sac (CSA) (mm^2^) was measured by tracing the outline of the thecal sac at the mid-axial slice at each disc level [7].

### 2.4. Clinical Outcome Assessments (COAs)

The participants completed the following clinical outcome assessments (COAs) preoperatively (Pre) and 12 months postoperatively (Post 12M).

#### 2.4.1. Visual Analog Scale

The degree of LBP, pain in the buttocks and lower limbs (leg pain), and numbness in the buttocks and lower limbs (leg numbness) were evaluated using a visual analogue scale (VAS) of 0–100 mm [8,9,10].

#### 2.4.2. Japanese Orthopaedic Association Back Pain Evaluation Questionnaire

The Japanese Orthopaedic Association Back Pain Evaluation Questionnaire (JOABPEQ) measures multidimensional conditions of low back disorders, including pain intensity, disability, and quality of life (QOL) [8,10,11]. It comprises 25 items across five subscales: LBP, lumbar function, walking ability, social life function, and mental health (seven items). The range of the score for each domain is independently evaluated from 0 to 100, with higher scores indicating a better condition.

#### 2.4.3. Oswestry Disability Index

The Oswestry Disability Index (ODI) is a questionnaire mostly used to assess LBP-related QOL, with higher scores indicating a worse condition. The ODI consists of 10 items that assess the level of pain and physical activity, including sleep, self-care, sexual life, social life, and traveling [12]. The Japanese version of the ODI, which has high reliability and validity [13], was used in this study. The total score, excluding the item on sexual life, was analysed in this study. Higher scores indicate a worse condition.

#### 2.4.4. Roland–Morris Disability Questionnaire

The Roland–Morris Disability Questionnaire (RDQ) assesses the degree of disability experienced during daily activities because of LBP [14]. The RDQ comprises 24 items that ask about the degree of disability experienced during daily activities, such as standing, walking, sitting, getting dressed, and working, with higher scores indicating a worse condition. The reliability, validity, and responsiveness of the Japanese version of the RDQ have been reported [15].

### 2.5. Statistical Analyses

The consistency of the disc area measured by CT and MRI was assessed using Pearson’s correlation coefficient test and intraclass correlation coefficient (ICC) using 35 randomly selected disc levels. The intra- and inter-rater reliabilities of the %EAD measurements were evaluated by ICC using 35 randomly selected disc levels, each measured in duplicate by two independent observers at different time points.

The %EAD data for each disc level were used to assess the association with MRI parameters. The mean %EAD of total disc levels (L1/L2 to L5/S1) was used to assess the association with age and anthropometric measurements, and the mean %EAD at surgical (decompression) levels was used to assess the association with COAs.

Differences in COAs, including VAS (mm) and the five domains of JOABPEQ, ODI, and RDQ, between the Pre and Post 12M periods were analysed using a paired T-test or Mann–Whitney U test. Correlations between %EAD and age, body height, body weight, BMI, MRI parameters, or pre- and postoperative COAs were evaluated using the Pearson correlation coefficient or Spearman rank-order correlation test. A multiple regression analysis was performed to identify factors contributing to %EAD as a dependent variable with COAs at Post 12M. Differences in COAs between the %EAD <30 and ≥30 groups were analysed using an unpaired T-test or Mann–Whitney U test Pre and Post 12M. Binary logistic regression was used to determine the factors associated with %EAD with COAs at Post 12M. Data are expressed as mean ± standard deviation. Statistical significance was set at *p* < 0.05. All statistical analyses were performed using the IBM SPSS Statistics software (version 28.0; IBM Japan, Tokyo, Japan).

## 3. Results

### 3.1. Patient Characteristics

In total, 63 patients were included in this study. The anthropometric measurements of all the patients were as follows: mean body height, 162.5 ± 7.3 cm; mean body weight, 65.6 ± 13.2 kg; and mean BMI, 24.8 ± 8.6 kg/m^2^. Posterior decompression was performed at L1/L2 in 8 patients (8 discs), L2/L3 in 29 patients (29 discs), L3/L4 in 45 patients (45 discs), L4/L5 in 38 patients (38 discs), and L5/S1 in 3 patients (3 discs). Posterior decompression was performed at the one-disc level in 22 (34.9%) patients, two-disc levels in 23 (36.5%) patients, three-disc levels in 17 (27.0%) patients, and four-disc levels in one (1.6%) patient.

The COAs Pre and Post 12M are summarized in Table 1. The mean preoperative VAS scores for LBP, leg pain, and leg numbness, ODI score, and RDQ score significantly decreased Post 12M (*p* < 0.01). The mean scores of the five domains of the JOABPEQ significantly increased Post 12M (*p* < 0.01).

### 3.2. Reliability of the Percentage of the Extended Area of the Disc against the Endplate Area (%EAD) Measurement

**Figure 3:**
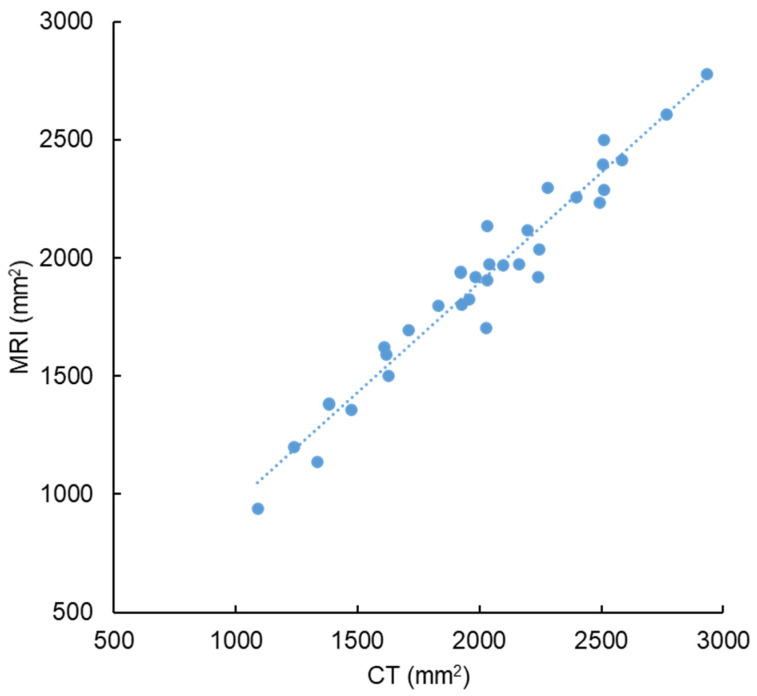
Consistency of disc area measured using computed tomography and magnetic resonance imaging.

The consistency of disc area measured between CT and MRI was excellent (r = 0.975, *p* < 0.01; mean ICC = 0.971 [0.947–0.984]) (Figure 3). The intra- and inter-rater reliabilities of the %EAD measurements were excellent, with ICC scores of 0.993 [0.987–0.997] and 0.964 [0.917–0.983], respectively.

### 3.3. Morphometry of Disc Bulging

**Figure 4:**
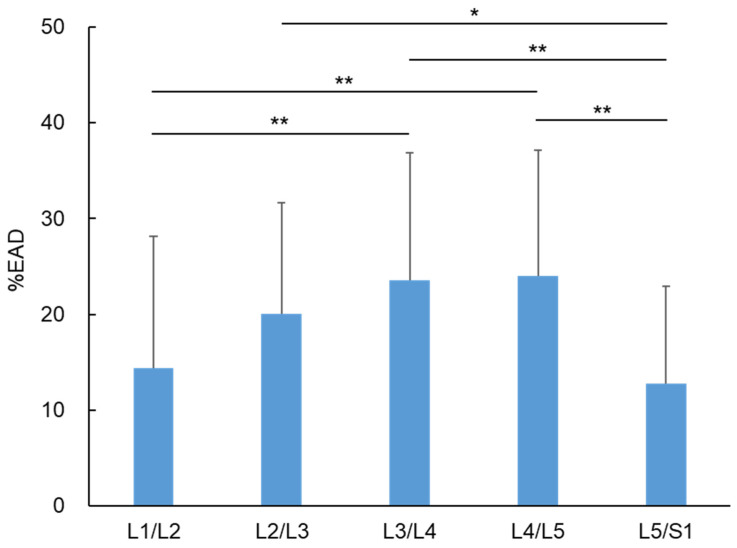
Percentage of the extended area of the disc against the endplate area (%EAD) at each disc level. %EAD at L1/L2 to L5/S1 is shown. * *p* < 0.05, ** *p* < 0.01.

Disc bulging at each disc level was evaluated using %EAD. The mean %EAD of 315 IVDs was 18.9 ± 8.0. The %EAD was highest at L4/L5 (24.0 ± 13.1), followed by L3/L4 (23.5 ± 13.3), L2/L3 (20.0 ± 11.6), L1/L2 (14.4 ± 13.7), and L5/S1 (12.8 ± 10.2). The %EAD at L3/L4 and L4/L5 was significantly higher than that at L1/L2 and L5/S1 (Figure 4).

**Figure 5:**
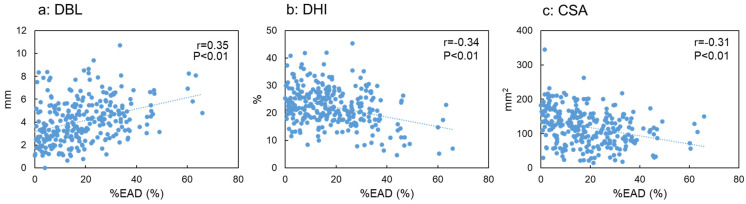
Correlation between the percentage of the extended area of the disc against the endplate area (%EAD) and magnetic resonance imaging measurements. The correlation between %EAD and disc bulging length (**a**), disc height index (**b**), and cross-sectional area of the thecal sac (**c**) is shown. r, correlation coefficient.

%EAD by disc level showed a significant but mild correlation with DBL (r = 0.35, *p* < 0.01, Figure 5a) and a significant negative correlation with DHI (r = −0.34, *p* < 0.01, Figure 5b) and CSA (r = −0.31, *p* < 0.01, Figure 5c). The mean %EAD (L1/L2 to L5/S1) was significantly but weakly correlated with body height (r = −0.27, *p* < 0.05). However, there were no significant differences between %EAD and age (r = 0.04, *p* = 0.64), body weight (r = −0.13, *p* = 0.30), or BMI (r = −0.03, *p* = 0.85).

### 3.4. Correlation between %EAD and COAs

The %EAD of the surgical (decompression) level (*n* = 123, 25.4 ± 12.4) was significantly higher than that of the nonsurgical level (*n* = 192, 14.8 ± 12.0) (*p* < 0.01). No significant correlations were found between %EAD and any preoperative COAs (Table 2). However, %EAD was significantly correlated with lumbar function, walking ability, and social life function domains of the JOABPEQ, ODI, and RDQ Post 12M (Table 2). %EAD showed a significant negative correlation with the postoperative change of the walking ability domain of the JOABPEQ (r = −0.319, *p* < 0.05) (Table 2).

The multiple regression analysis revealed that %EAD was significantly associated with the postoperative score of the walking ability domain of the JOABPEQ (Table 3).

### 3.5. Correlation between Disc Bulging Length and COAs

The mean of the total DBL was 4.0 ± 1.3 (mm). The DBL of the surgical (decompression) level (*n* = 123, 5.2 ± 1.5) was significantly higher than that of the nonsurgical level (*n* = 192, 3.4 ± 1.7) (*p* < 0.01). There were no significant correlations between DBL and any COAs Pre and Post 12M.

### 3.6. Association between %EAD Classification and COAs

**Figure 6:**
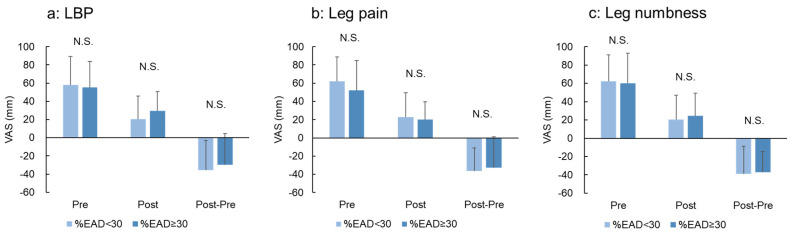
Visual analogue scale (VAS) according to the extent of percentage of the extended area of the disc against the endplate area (%EAD). The patients were divided into two groups according to the extent of the mean %EAD at the surgical (decompression) level (%EAD <30 and ≥30 groups). The degrees of (**a**) low back pain (LBP), (**b**) pain in the buttocks and lower limbs (leg pain), and (**c**) numbness in the buttocks and lower limbs (leg numbness) were evaluated using a VAS of 0−100 mm. The mean VAS scores of each group in the Pre and Post 12M periods and the change (Post 12−Pre) are presented.

There were no significant differences in preoperative VAS scores for LBP, leg pain, and leg numbness between the %EAD <30 and ≥30 groups (Figure 6a–c). No significant differences in the postoperative VAS scores were found between the groups (Figure 6a–c). No significant differences were identified in the postoperative changes (Post 12M—Pre) in the VAS for LBP, leg pain, and leg numbness between the groups (Figure 6a–c).

**Figure 7:**
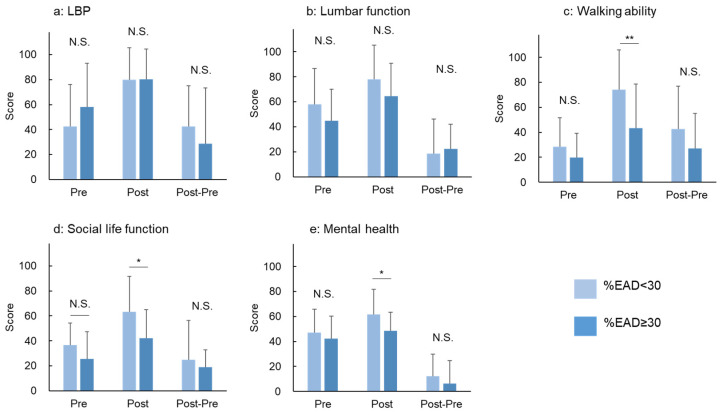
The Japanese Orthopaedic Association Back Pain Evaluation Questionnaire (JOABPEQ) according to the extent of the percentage of the extended area of the disc against the endplate area (%EAD). The patients were divided into two groups according to the extent of mean %EAD at surgical (decompression) levels (%EAD <30 and ≥30 groups). The JOABPEQ is composed of 25 items across five subscales: (**a**) low back pain, (**b**) lumbar function, (**c**) walking ability, (**d**) social life function, and (**e**) mental health. The mean score of each group at the Pre and Post 12M periods and change (Post 12—Pre) are presented. * *p* < 0.05, ** *p* < 0.01.

There were no significant differences in the preoperative scores of all the five domains of the JOABPEQ between the %EAD <30 and ≥30 groups (Figure 7a–e). There were no significant differences in the scores of the LBP and lumbar function domains Post 12M between the groups (Figure 7a,b). However, the scores for walking ability, social life function, and mental health domains Post 12M in the %EAD <30 group were significantly higher than those in the %EAD ≥30 group (Figure 7c–e). No significant differences were identified in the postoperative changes (Post 12M—Pre) of all five domains of the JOABPEQ between the groups (Figure 7a–e).

**Figure 8:**
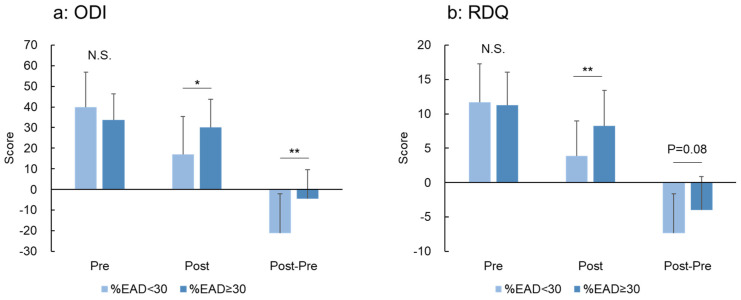
Oswestry Disability Index (ODI) (**a**) and Roland–Morris Disability Questionnaire (RDQ) (**b**) scores according to the extent of the percentage of the extended area of the disc against the endplate area (%EAD). The patients were divided into two groups according to the extent of mean %EAD at surgical (decompression) levels (%EAD <30 and ≥30 groups). The mean score of each group at the Pre and Post 12M periods and change (Post 12—Pre) are presented. * *p* < 0.05, ** *p* < 0.01.

The preoperative ODI and RDQ scores did not differ significantly between the %EAD <30 and ≥30 groups. The ODI and RDQ scores Post 12M were significantly higher in the %EAD ≥30 group compared with the %EAD <30 group (*p* < 0.05 and *p* < 0.01, respectively) (Figure 8a,b). The postoperative change (Post 12M—Pre) of ODI scores was significantly lower in the %EAD ≥30 group compared with that of the %EAD <30 group (Figure 8a). The postoperative change in the RDQ score was also lower in the %EAD ≥30 group compared with that in the %EAD <30 group; however, the difference was not significant (*p* = 0.08, Figure 8b).

The logistic regression analysis revealed that the %EAD ≥30 group was significantly associated with a lower score of the walking ability domain of the JOABPEQ Post 12M (Table 4).

## 4. Discussion

The current study quantitatively evaluated the morphology of disc bulging using axial CT-MPR. In this study, disc bulging was defined as the percentage of the EAD beyond the endplate against the endplate area and was termed %EAD. The outer margin of the IVD tissue in the axial CT plane was determined at the midline of the craniocaudal endplate by setting a definite CT threshold value that was applied to all samples. High homology was confirmed between the disc areas determined using CT-MPR and T2-weight imaging MRI, suggesting that the CT threshold value set in this study successfully extracts the axial disc area.

The results of the current study showed that %EAD was negatively correlated with CSA, suggesting that an increase in %EAD is associated with an increased severity of lumbar canal stenosis. In addition, the current study revealed that the increase in %EAD significantly correlated with disc height narrowing, which represents a structural change along with IVD degeneration [6].

In contrast, looking at the correlation with the physical findings, the mean %EAD (of the whole lumbar spine) was not correlated with body weight and BMI; however, it was negatively correlated with body height. Because %EAD was negatively correlated with DHI, degenerative scoliosis and disc height narrowing of the lumbar spine along with disc bulging are factors associated with decreased body height in patients with LSS [16].

Next, we evaluated the association between disc bulging and clinical symptoms and surgical outcomes in patients with LSS. Interestingly, %EAD did not correlate with any preoperative COAs; however, it was significantly correlated with postoperative COAs, including the JOABPEQ (lumbar function, walking ability, social life function domains), ODI, and RDQ scores. The multiple regression analysis revealed that %EAD was significantly associated with the walking ability domains of the JOABPEQ Post 12M. Similarly, when %EAD was classified into two groups with a border setting of 30%, no significant differences in any of the preoperative COAs were identified between the two groups. However, the walking ability, social life function, and mental health domains of the JOABPEQ, ODI, and RDQ were significantly worse in the %EAD ≥30 group than in the %EAD <30 group Post 12M. Finally, the logistic regression analysis also revealed that the %EAD ≥30 group was significantly associated with a worse walking ability domain of the JOABPEQ Post 12M.

These results suggest that disc bulging evaluated using %EAD shows no significant association with preoperative neurological symptoms and/or LBP-related QOL, resulting in a statistically significant association after decompression. Namely, the release of nervous and/or vascular elements within the lumbar spinal canal by posterior decompression surgery significantly improves neurological symptoms; meanwhile, the effect of disc bulging on LBP-related QOL may become noticeable. In contrast, DBL, which represents the distance of the posterior protrusion of disc bulging, did not have significant effects on COAs Pre and Post 12M. This may imply that the extent of building into the spinal canal alone does not affect pre- and postoperative COAs.

Disc bulging is one of these findings, along with the progression of disc degeneration, and is often associated with LBP [17,18]. Furthermore, disc bulging in the lumbar spine affects spinal function, including lumbar sagittal and coronal imbalance [16]. Liu et al. [19] reported that disc bulging and lumbar lordosis were correlated with fatty infiltration of the multifidus muscle, which was associated with functional failure of the lumbar spine in patients with LSS. Therefore, the presence of disc bulging, as evaluated by the %EAD after decompression surgery, is associated with worse LBP-related QOL in patients with LSS.

This study has some limitations. First, the number of patients included was small. Second, lumbar sagittal and coronal imbalances, which may affect COAs Pre and Post 12M, were not evaluated in this study. Patients who underwent decompression surgery but not fusion surgery were included in this study; however, segmental instability, which may have an effect on postoperative outcomes, was not evaluated. Finally, the current preliminary study, for the first time, quantitatively evaluates the morphology of lumbar disc bulging using CT images by %EAD. Therefore, it is essential to further assess the clinical significance of %EAD by increasing the number of patients with degenerative lumbar diseases in future studies.

## 5. Conclusions

This study quantitatively evaluated disc bulging using %EAD as an indicator in patients with LSS. The results showed that disc bulging evaluated by %EAD was significantly correlated with the extent of disc height narrowing and canal stenosis. On the other hand, %EAD showed no significant correlation with lower extremity neurological symptoms and LBP-related QOL preoperatively. However, the patients whose %EAD was more than 30% showed worse LBP-related, especially that related to walking ability, after decompression surgery. Therefore, spinal surgeons should consider that the presence of disc bulging has negative effects on poor surgical outcomes after decompression surgery in patients with LSS, even without segmental instability.

## Figures and Tables

**Table 1:** Preoperative (Pre) and 12-month postoperative (Post 12M) clinical outcome assessments (COAs).

	Pre	Post 12M
VAS in LBP	57.0 (31.8)	22.9 (24.9) **
VAS in leg pain	57.8 (28.5)	22.3 (25.2) **
VAS in leg numbness	60.3 (30.8)	22.1 (26.0) **
LBP of the JOABPEQ	42.2 (32.9)	80.9 (24.5) **
Lumbar function of the JOABPEQ	54.6 (27.7)	74.3 (27.7) **
Walking ability of the JOABPEQ	26.8 (22.5)	65.3 (35.3) **
Social life function of the JOABPEQ	34.2 (20.2)	57.4 (28.9) **
Mental health of the JOABPEQ	47.6 (19.1)	58.1 (19.9) **
ODI (%)	37.6 (16.4)	20.7 (18.4) **
RDQ	11.6 (5.0)	5.1 (5.4) **

The degrees of low back pain (LBP), leg pain, and leg numbness were evaluated using a visual analogue scale (VAS) of 0–100 (mm). The five domains of the Japanese Orthopaedic Association Back Pain Evaluation Questionnaire are low back pain, lumbar function, walking ability, social life function, and mental health. ODI, Oswestry Disability Index. Numbers in parentheses indicate standard deviations. ** *p* < 0.01 vs. Pre.

**Table 2:** Correlation between the %EAD and clinical outcome assessments (COAs).

	VAS			JOABPEQ					ODI (%)	RDQ
	LBP	Leg Pain	Leg Numbness	Low Back Pain	Lumbar Function	Walking Ability	Social Life Function	Mental Health		
Pre	−0.102	−0.098	0.074	0.062	−0.255	−0.029	−0.133	−0.053	−0.045	0.006
Post 12M	0.110	−0.017	0.125	−0.027	−0.372 **	−0.466 **	−0.335 **	−0.263	0.340 *	0.354 *
Change	0.097	0.012	−0.044	−0.035	−0.016	−0.319 *	−0.159	0.168	−0.271	0.160

The degrees of low back pain (LBP), pain in the buttocks and lower limbs, and numbness in the buttocks and lower limbs were evaluated using a visual analogue scale (VAS) of 0–100 (mm). The Japanese Orthopaedic Association Back Pain Evaluation Questionnaire is composed of 25 items across five domains: LBP, lumbar function, walking ability, social life function, and mental health. The five domains of the JOA Back Pain Evaluation Questionnaire are low back pain, lumbar function, walking ability, social life function, and mental health. ODI, Oswestry Disability Index; RDQ, Roland–Morris Disability Questionnaire. Change: preoperative (Pre) COAs to 12-month postoperative (Post 12M) COAs. The number of each cell indicates the correlation coefficient (r). ** *p* < 0.01, * *p* < 0.05.

**Table 3:** Association between %EAD and clinical outcome assessments (COAs) 12 months postoperatively.

	β	βstand	t	*p* Value
Walking ability	−0.001	0.000	−2.353	<0.05

Walking ability is a domain of the Japanese Orthopaedic Association Back Pain Evaluation Questionnaire. Standardized coefficients: βstand. R2: 0.11, *p* < 0.05.

**Table 4:** Association between %EAD classification and clinical outcome assessments 12 months postoperatively.

Variable	OR	95% CI	*p*-Value
Walking ability	1.658	0.956–0.994	0.011

Walking ability is a domain of the Japanese Orthopaedic Association back pain evaluation questionnaire. OR, odds ratio; CI, confidence interval.

## Data Availability

The data presented in this study are available on request from the corresponding author.
